# Comparison of Fine Structure of the Compound Eyes in *Eucryptorrhynchus scrobiculatus* and *Eucryptorrhynchus brandti* Adults

**DOI:** 10.3390/insects14080699

**Published:** 2023-08-10

**Authors:** Yingying Hao, Qi Wang, Chao Wen, Junbao Wen

**Affiliations:** 1 State Key Laboratory of Efficient Production of Forest Resources, Beijing Forestry University, Beijing 100083, China; haoyying@bjfu.edu.cn (Y.H.); wangqi77@bjfu.edu.cn (Q.W.); 2 Beijing Key Laboratory for Forest Pest Control, Beijing Forestry University, Beijing 100083, China; 3 College of Forestry, Beijing Forestry University, Beijing 100083, China; 4 School of Grassland Science, Beijing Forestry University, Beijing 100083, China

**Keywords:** vision, apposition, dark/light adaptation, weevil

## Abstract

**Simple Summary:**

*Eucryptorrhynchus scrobiculatus* and its related species *Eucryptorrhynchus brandti* together damage *Ailanthus altissima* and *Ailanthus altissima* ‘Qiantou’. *E. scrobiculatus* possesses a large compound eye area and a higher number of ommatidia than *E. brandti*. Each ommatidium of *E. scrobiculatus* and *E. brandti* consists of a cornea, a crystalline cone, eight retinal cells, and its semi-fused rhabdom. The internal structure, including the cornea and rhabdom, of *E. scrobiculatus* is larger than that of *E. brandti*. Light/dark adaptational changes affect cone length, the position of pigment grains, and the cross-sectional area of the rhabdoms.

**Abstract:**

*Eucryptorrhynchus scrobiculatus* and *E. brandti* are the main borers of *Ailanthus altissima*, causing serious economic and ecological losses. The external morphology and internal ultrastructure of the compound eyes of two related weevils were investigated with light microscopy, scanning electron microscopy, and transmission electron microscopy. *E. scrobiculatus* and *E. brandti* possess a pair of reniform apposition compound eyes and contain about 550 ommatidia per eye. The interommatidial angle of *E. scrobiculatus* and *E. brandti* are 7.08 ± 0.31° and 4.84 ± 0.49°, respectively. The corneal thickness, rhabdom length, and ommatidium length of *E. scrobiculatus* are significantly greater than those of *E. brandti*. Under light-adapted conditions, the pigment granules are mainly distributed at the junction of the cone and the rhabdom, and the diameter and the cross-sectional area of the middle end of the rhabdom is increased in the two weevil species. Under dark-adapted conditions, the pigment granules shift longitudinally and are evenly distributed on both sides of the cone and the rhabdom, and the diameter and cross-sectional area of the middle end of the rhabdom are decreased. The discrepancy in visual structure is beneficial for adaptation to niche differentiation of the two related species. The present results suggest that the two weevils possess different visual organ structures to perceive visual information in the external environment.

## 1. Introduction

Studying insect behavior characteristics and their associated morphological structure is beneficial for understanding pest occurrence [1]. The compound eye of insects is the main visual organ for detecting and recognizing the external environment, which affects the ability of insects to recognize shapes and colors, as well as detect specific objects in the surrounding environment [2,3,4,5]. The structure and light-sensitive properties of compound eyes vary among different insect species, which are related to their behavioral habits. In-depth studies of the differences in compound eye structure are beneficial for investigating the mechanisms by which insects’ visual perception regulates their behavior [6]. Although the study of the structure and function of the compound eye has long been emphasized, only some studies have been focused on two related species. Studying the compound eye structure of two closely related species may provide evidence to explain their behavioral differences.

Compound eyes consist of a host of units called ommatidia, and each ommatidium consists of a corneal lens, a crystalline cone, reticular cells, and its rhabdom [7]. The number of ommatidia may relate to insect vision regarding sensitivity, resolution, and visual field [8]. There are some differences in the internal structure of the compound eyes between nocturnal and diurnal insects, with nocturnal insects mainly possessing the superposition eye type and diurnal insects possessing the apposition eye type [9]. Nocturnal insects can distinguish colors accurately, fly freely, and navigate and locate themselves with blurred moonlight [10]. The dynamic changes in the ommatidial structure during dark/light adaptation suggest that the eye attempts to optimize its vision in response to different levels of ambient illumination [11]. Morphological changes in dioptric apparatus and rhabdoms, as well as the movement of screening pigment granules to regulate the amount of light that reaches the photoreceptive layer [12,13], are adaptations to variation in light and darkness in the environment.

*Ailanthus altissima* is a roadside tree species that has strong adaptability to adverse climates and is resistant to salt, alkali, drought, and dust, which has made outstanding contributions to the ecological environment [14]. *Eucryptorrhynchus scrobiculatus* and *E. brandti* are the main borer pests of *A. altissima*, weakening plants and even causing mortality [15,16,17]. *E. brandti* adults are approximately 11.5 mm long and 4.6 mm wide, while *E. scrobiculatus* adults are 10–15 mm long and 8.5 mm wide [18]. The trophic niches of *E. scrobiculatus* and *E. brandti* adults differ significantly. While *E. brandti* adults primarily eat the stem, *E. scrobiculatus* adults primarily eat 1-year-old branches, perennial branches, and petioles [19]. When *A. altissima* sprouts blossom in the middle of April, the overwintering adults of *E. scrobiculatus* are discovered, providing enough food for their additional nourishment [20]. Although there is no spatial niche differentiation between *E. scrobiculatus* and *E. brandti* in the large-scale space of China, there is a spatial niche differentiation between the two species in the tree. *E. scrobiculatus* adults are most common between 0 cm (ground surface) and 250 cm. On *A. altissima*, the vertical distribution of *E. brandti* adults is primarily found between 0.1 and 50 cm and over 250 cm [19]. According to the niche theory, when species are in an ecosystem with limited resources, there will frequently be severe interspecific competition among them, leading to the extinction of the species with weak competitive capacity. However, some ecologically similar species may coexist, and ecological niche differentiation occurs to alleviate or avoid such fierce competition [21]. There is a significant amount of trophic niche differentiation and a small amount of spatial niche differentiation between the two weevil adults [19]. Opsin is a photoreceptor in the compound eye of insects and plays an important role in phototaxis [22]. The selection of different wavelengths of light differs between *E. scrobiculatus* and *E. brandti*, and the attraction index of different wavelengths of light to *E. scrobiculatus* and *E. brandti* changes after light and dark treatments [23]. Both related weevils can be attracted by light, but only *E. scrobiculatus* can perceive polarized light [24]. We hypothesize that the differentiation of ecological niches between *E. scrobiculatus* and *E. brandti* might be related to variation in the structure of the visual organ. This study compared the compound eye structures of the two related weevils with different habitats.

## 2. Materials and Methods

### 2.1. Insect Source

Adult males and females of *E. scrobiculatus* and *E. brandti* were collected in mid-May 2022 from Lingwu City, Ningxia Hui Autonomous Region, China. The collected insects were placed in gauze bags (30 × 30 cm) in incubators at 26 ± 1 °C, 60–70% relative humidity, and a photoperiod of 14:10 h (L:D). The adults were reared with branches and leaves of *A. altissima*.

To conduct light/dark adaption, the female adults were placed in the light conditions (10,000 Lux) of the incubator for 12 h (light-adapted) or in the dark conditions of the incubator for 12 h (dark-adapted) before fixation. The light-adapted insects were decapitated and fixed during the daytime, while dark-adapted specimens were decapitated and fixed in dark conditions.

### 2.2. Scanning Electron Microscopy (SEM)

For scanning electron microscopy (SEM), ten (five males and five females) *E. scrobiculatus* and *E. brandti* adults were used. After decapitation, compound eyes were immediately fixed in 2.5% glutaraldehyde for at least 24 h. They were then rinsed three times with 0.1 M PBS (PH = 7.2), each time for 10 min. Then, the head was dehydrated under an alcohol gradient (10 min in 50%, 60%, 70%, 75%, 85%, and then in 95% and 100% for 30 min). After air drying for 24 h, the specimens were sputter-coated with gold at 30 mA current for 100 s (Hitachi MC1000, Tokyo, Japan). A scanning electron microscope (Hitachi SU8010, Japan) was used to take micrographs of the eye surface.

### 2.3. Transmission Electron Microscopy (TEM)

For transmission electron microscopy (TEM) analysis, dissected compound eye samples were fixed in 2% paraformaldehyde and 2.5% glutaraldehyde and placed in storage at 4 °C for more than 24 h. Samples were washed 4 times with 0.1 M cacodylate buffer (pH = 7.2) for 15 min. Specimens were post-fixed in 1% OsO4 for 24 h, washed 3 times with ddH2O for 15 min each, then placed in 1% uranyl acetate and left for 1 h at room temperature. Samples were washed 4 times with ddH_2_O each time for 15 min. Compound eyes were dehydrated in an alcohol series (30%, 50%, 70%, 80%, 90%, each time 20 min), 100% alcohol (4 times) and propylene oxide (two times). Specimens were infiltrated with different 812 resin/propylene oxide ratios (3:1, 1:1, 1:3, pure 812 resin) for 8 h. They were then permeated twice in pure 812 resin, each for 8 h, and finally hardened at 60 °C for 24 h. The embedded samples were cut into 80 nm thickness ultrathin sections using the frozen ultrathin sectioning machine (Leica EM UC7, Switzerland, Germany). The sections were stained with 2% aqueous uranyl acetate for 15 min and observed in the transmission electron microscope (Hitachi-7650, Tokyo, Japan) at 80 kV.

### 2.4. Light Microscopy (LM)

For light microscopy (LM), translucent sections of approximately 500 nm thickness were cut with a microtome (Leica EM UC7, Switzerland, Germany). These sections were stained on a hot plate with 1% aqueous toluidine blue for 100 s. The semi-thin sections were sealed with clear nail polish. Micrographs were taken with light microscopy (Pannoramic Scan, Budapest, Hungary).

### 2.5. Data Analysis

Scanning electron microscopy (SEM) was used to determine the compound eye area, the number of hexagonal and irregular facets, the area of facets, and facet diameter. The longitudinal sections of LM were used to measure the dimensions of the corneal lens, cones, rhabdoms, ommatidium, and interommatidial angle of the ommatidium. LM transverse sections were used to measure the diameter and cross-sectional area of the rhabdom. TEM was used to measure the number of retinal cells and the diameter of the rhabdomere microvilli. All measurements were performed using Fiji-ImageJ (version 1.4) and CaseViewer software (version 2.6). Data were counted using SPSS software (version 26.0), independent samples *t*-test, and one-way ANOVA with Tukey multiple comparisons.

## 3. Results

### 3.1. External Morphology of the Compound Eyes of E. scrobiculatus and E. brandti

**Figure 1:**
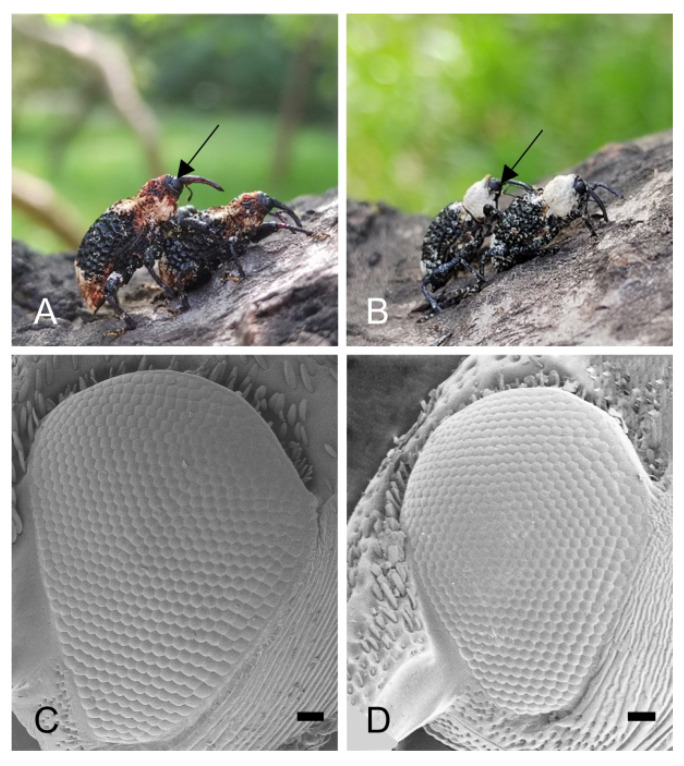
The external morphology of the compound eyes of *E. scrobiculatus* and *E. brandti* adults. (**A**) *E. scrobiculatus* adults: position of compound eye indicated by the arrow. (**B**) *E. brandti* adults: position of compound eye indicated by the arrow. (**C**) Scanning electron micrograph of the lateral view of the compound eye of *E. scrobiculatus*. (**D**) Scanning electron micrograph of the lateral view of the compound eye of *E. brandti*. Scale bars: (**C**,**D**) = 100 μm.

**Figure 2:**
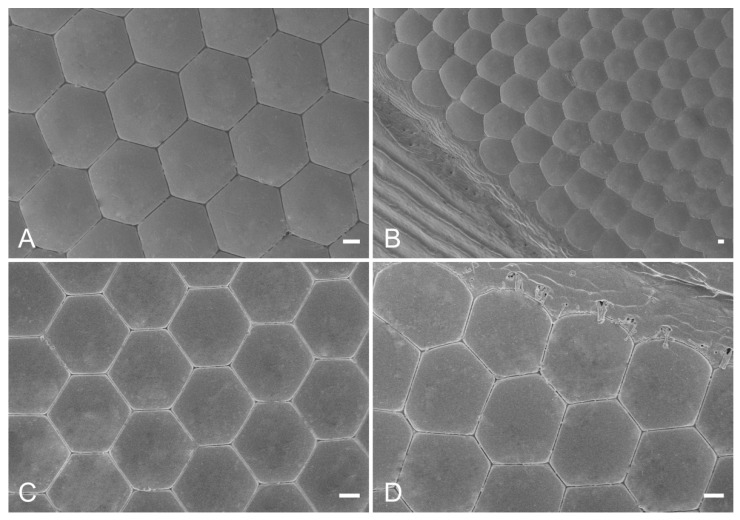
The scanning electron microscope of the compound eyes of *E. scrobiculatus* and *E. brandti*. (**A**) A close-up micrograph of the array of hexagonal facets of *E. scrobiculatus*. (**B**) A close-up micrograph with irregularly shaped facets of *E. scrobiculatus* near the edge of the eye. (**C**) A close-up micrograph of the array of hexagonal facets of *E. brandti*. (**D**) A close-up micrograph with some irregularly shaped facets of *E. brandti* near the edge of the eye. Scale bars: (**A**–**D**) = 10 μm.

Both *E. scrobiculatus* (Figure 1A) and *E. brandti* (Figure 1B) have a pair of reniform compound eyes (Figure 1C,D) located on either side of the symmetrical head. The compound eyes of both *E. scrobiculatus* and *E. brandti* are composed of ommatidia with slightly convex faces; most ommatidia are hexagonal, with occasional irregular compound eyes at the margins (Figure 2A–D). The diameter of the hexagonal facets of female and male *E. scrobiculatus* adults were 50.67 ± 1.41 μm and 52.09 ± 1.43 μm, respectively, with no significant difference between male and female adults (*p* > 0.05, Table 1); the diameter of the hexagonal eye facets of female and male adult *E. brandti* were 44.14 ± 0.69 μm and 41.69 ± 0.67 μm, respectively, with no significant difference between male and female adults (*p* > 0.05, Table 1). In contrast, the diameter of the hexagonal eyes of *E. scrobiculatus* was significantly larger than *E. brandti* (*p* < 0.05, Table 1). The number of ommatidia of female and male *E. scrobiculatus* was 561 ± 7 and 554 ± 5, respectively, with no significant difference between male and female adults (*p* > 0.05, Table 1); the number of ommatidia of female and male *E. brandti* was 538 ± 11 and 511 ± 7, respectively, with no significant difference between male and female adults (*p* > 0.05, Table 1). The number of ommatidia was significantly higher in *E. scrobiculatus* than in *E. brandti*, whether female or male (*p* < 0.05, Table 1). The total compound eye area was 1.100 ± 0.042 mm^2^ and 1.025 ± 0.024 mm^2^ for female and male *E. scrobiculatus,* respectively, with no significant difference between male and female adults (*p* > 0.05, Table 1); the total compound eye area was 0.777 ± 0.043 mm^2^ and 0.647 ± 0.025 mm^2^ for female and male *E. brandti,* respectively, with no significant difference between male and female adults (*p* > 0.05, Table 1). The total compound eye area of female and male *E. scrobiculatus* was significantly more than that of male and female *E. brandti* (*p* < 0.05, Table 1). The interommatidial angle was 7.08 ± 0.31° and 4.84 ± 0.49° for *E. scrobiculatus* and *E. brandti*, respectively (Table 2). Both *E. scrobiculatus* and *E. brandti* have no corneal papillae or sensory hairs on the surface of the compound eye (Figure 2A–D).

### 3.2. Ultrastructure of the Compound Eyes in E. scrobiculatus and E. brandti Adults

**Figure 3:**
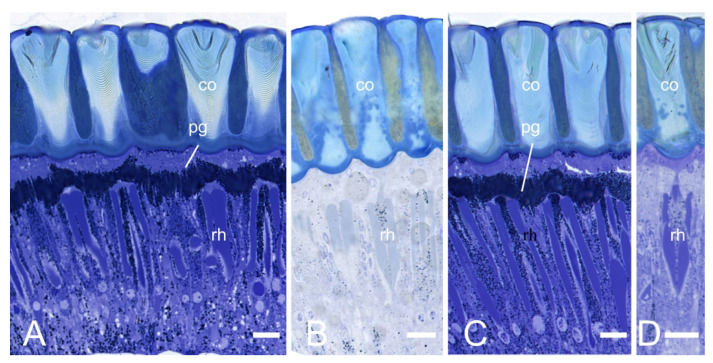
Light microscopy (LM) at different levels of the longitudinal section of the compound eye of *E. scrobiculatus* and *E. brandti* adults. (**A**) Sections of the compound eye of *E. scrobiculatus* in the light-adapted states, with the pigment particles distributed at the distal end of the rhabdom. (**B**) Sections of the compound eye of *E. scrobiculatus* in the dark-adapted states, with pigment grains evenly around the rhabdoms. (**C**) Sections of the compound eye of *E. brandti* in the light-adapted states. (**D**) Sections of the compound eye of *E. brandti* in the dark-adapted states. co, cornea; rh, rhabdom; pg, pigment particles. Scale bars: (**A**–**D**) = 20 μm.

**Figure 4:**
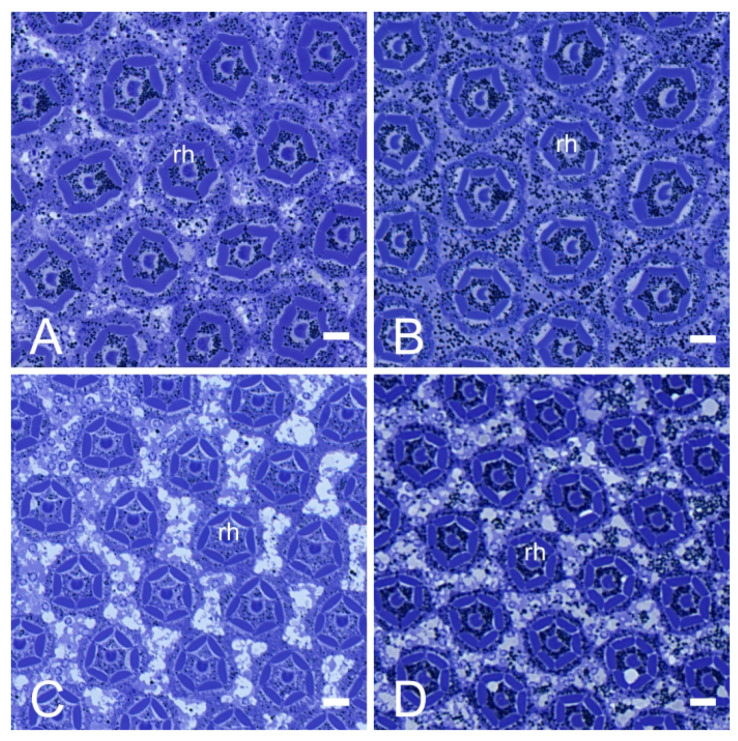
Light microscopy (LM) at different levels of the transverse section of the compound eye of *E. scrobiculatus* and *E. brandti* adults. (**A**) Sections of the eye of *E. scrobiculatus* in the light-adapted states. (**B**) Sections of the eye of *E. scrobiculatus* in the dark-adapted states. (**C**) Sections of the eye of *E. brandti* in the light-adapted states. (**D**) Sections of the eye of *E. brandti* in the dark-adapted states. rh, rhabdom. Scale bars: (**A**–**D**) = 10 μm.

**Figure 5:**
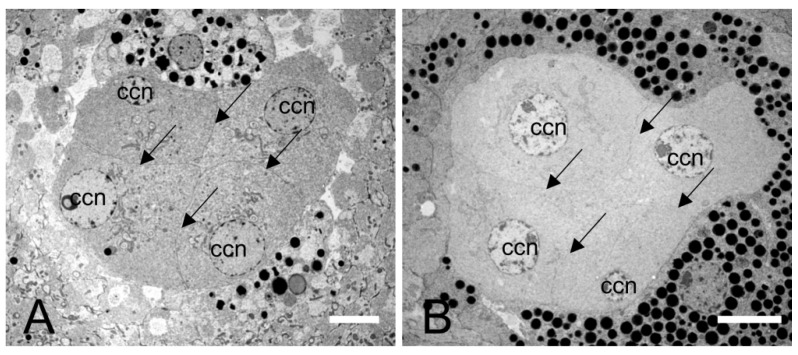
Transmission electron microscope of the crystal cone cells of *E. scrobiculatus* and *E. brandti*. (**A**) Transverse section of the cone cells of *E. scrobiculatus*. (**B**) Transverse section of the cone cells of *E. brandti*. ccn, crystalline cone nucleus. The arrow points to the boundary of the cone cells. Scale bars: (**A**,**B**) = 5 μm.

**Figure 6:**
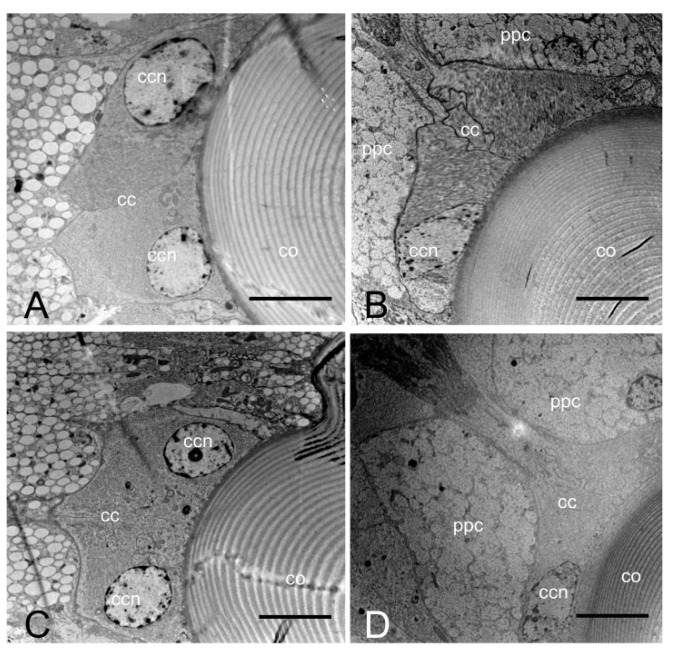
Transmission electron microscopic longitudinal section of the cone cells of *E. scrobiculatus* and *E. brandti*. (**A**) Section of crystal cone cells of *E. scrobiculatus* in the light-adapted state. (**B**) Section of crystal cone cells of *E. scrobiculatus* in the dark-adapted state. (**C**) Section of crystal cone cells of *E. brandti* in the light-adapted state. (**D**) Section of crystal cone cells of *E. brandti* in the dark-adapted state. Both *E. scrobiculatus* and *E. brandti* have longer cone cells in the light adaptation than in the dark adaptation. co, cornea; cc, crystalline cone; ccn, crystalline cone nucleus; ppc, primary pigment cell. Scale bars: (**A**–**D**) = 5 μm.

There is no clear zone between the crystalline cone and the rhabdom in the compound eye of *E. scrobiculatus* and *E. brandti* adults, which is typical of apposition eyes (Figure 3A–D). The ommatidia structure consists of a corneal lens, crystalline cone, pigment cells, rhabdom (Figure 4A–D), and basement membrane from the distal to the proximal end, in that order. The dioptric apparatus consists of corneal lenses and a crystalline cone. The corneal thickness of *E. scrobiculatus* and *E. brandti* is 99.16 ± 1.63 μm and 82.26 ± 0.86 μm, respectively, and the *E. scrobiculatus* cornea is significantly thicker than that of *E. brandti* (*p* < 0.01, Table 2). The crystalline cone is located below the cornea and formed by four cone cells, which are slightly rounded in cross-section (Figure 5A,B), and a significant number of pigment granules surround the crystalline cone. The crystalline cone cell nucleus is located in the distal part of the cell (Figure 6A–D).

**Figure 7:**
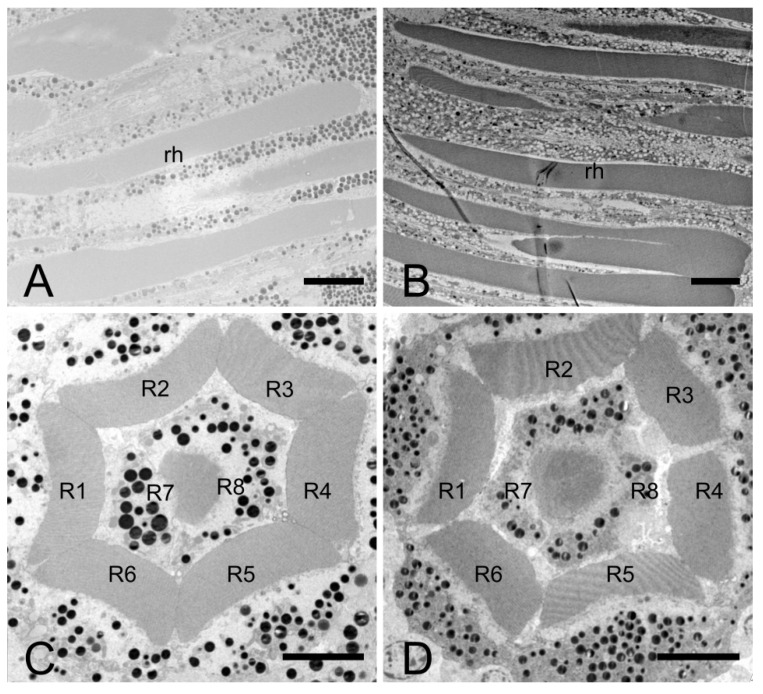
Transmission electron microscopic micrograph of the rhabdom of the ommatidia of female *E. scrobiculatus* and *E. brandti* showing photoreceptive layer consisting of eight retinular cells (R1 to R8). (**A**) Longitudinal section of the rhabdom of *E. scrobiculatus*. (**B**) Longitudinal section of the rhabdom of *E. brandti*. (**C**) Transverse section of the rhabdom of *E. scrobiculatus*. (**D**) Transverse section of the rhabdom of *E. brandti*. The length of *E. scrobiculatus*’s rhabdom and the cross-sectional area in the middle of the rhabdom are larger than those of *E. brandti.* rh, rhabdom. Scale bars: (**A**,**B**) = 10 μm, (**C**,**D**) = 5 μm.

**Figure 8:**
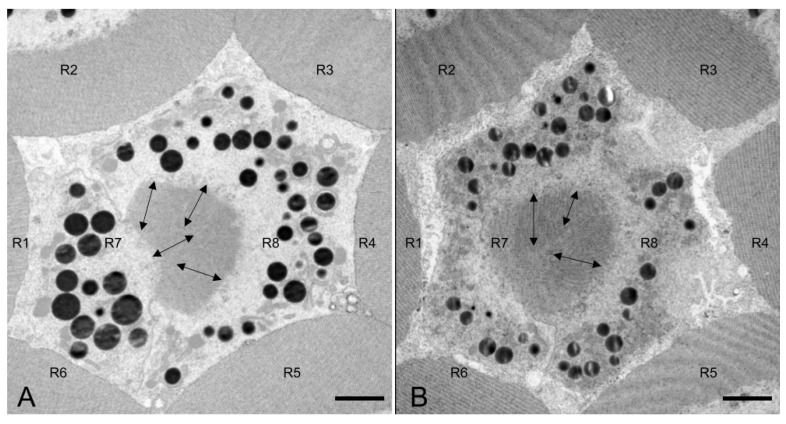
Transmission electron microscopic longitudinal section of the rhabdom of *E. scrobiculatus* and *E. brandti*. (**A**) Transverse section of the rhabdom of *E. scrobiculatus*. (**B**) Transverse section of the rhabdom of *E. brandti*. The arrow indicates the direction of the microvilli on the rhabdom. Scale bars: (**A**,**B**) = 2 μm.

The lengths of the rhabdom of *E. scrobiculatus* and *E. brandti* are 69.12 ± 2.22 μm and 59.08 ± 2.79 μm, respectively (Table 2). The photosensitive layers of both *E. scrobiculatus* and *E. brandti* are composed of eight retinal cells and their rhabdomeres (Figure 7A–D). The numbering of the retinal cells follows the method by Wachmann [25]. There are eight retinal cells in the ommatidium, two central cells (R7–R8), surrounded by six peripheral cells (R1–R6). The peripheral rhabdomeres are in contact with each other and have well-defined borders. Each rhabdomere consists of numerous microvilli, and those of *E. scrobiculatus* and *E. brandti* were 2.40 ± 0.20 μm and 1.76 ± 0.13 μm in diameter, respectively (Table 2). The distorted orientation of the rhabdom of *E. scrobiculatus* and *E. brandti* was not aligned in two orthogonal directions (Figure 8A,B). The ommatidium of *E. scrobiculatus* and *E. brandti* contains two primary pigment cells (PPCs) and an indeterminate number of secondary pigment cells (SPCs) (Figure 6B,D and Figure 7A,B). The primary pigment cells envelop each of the four cone cells. The cone cells (Figure 5A,B), PPCs, and SPCs all contained numerous spherical electron-dense and opaque screening pigment granules, as well as mitochondria in their cytoplasm.

### 3.3. Dark/Light Adaptational Changes of the Compound Eyes

The pigment granules of *E. scrobiculatus* and *E. brandti* are distributed at the distal end of the rhabdom in the light-adapted (LA) state (Figure 3A,C and Figure 4A,C). They are more evenly distributed in the dark-adapted (DA) state (Figure 3B,D and Figure 4B,D). In the LA state, the length of the cone cells (Figure 6A), the diameter, and the cross-sectional area of the rhabdom (Figure 4A) of *E. scrobiculatus* were 9.99 ± 0.38 μm, 24.18 ± 0.20 μm, and 436.08 ± 6.36 μm^2^, respectively (Table 3). In the DA state, the length of the cone cells (Figure 6B) and the diameter and the cross-sectional area of the rhabdom (Figure 4B) of *E. scrobiculatus* were 6.74 ± 0.48 μm, 21.21 ± 0.31 μm, and 372.31 ± 7.96 μm^2^, respectively (Table 3). There were significant differences in crystal cone cell length, rhabdom diameter, and rhabdom cross-sectional area in *E. scrobiculatus* under light/dark adaptation (*p* < 0.01, Table 3).

In the LA state, the length of the cone cells (Figure 6C), the diameter, and the cross-sectional area of the rhabdom (Figure 4C) of *E. brandti* were 7.53 ± 0.40 μm, 20.62 ± 0.23 μm, and 342.66 ± 3.93 μm^2^, respectively (Table 3). In the DA state, the length of the cone cells (Figure 6D), the diameter, and the cross-sectional area of the rhabdom (Figure 4D) of *E. brandti* were 4.50 ± 0.58 μm, 19.63 ± 0.17 μm, and 313.84 ± 4.30 μm^2^, respectively (Table 3). Significant differences were found in the length, rhabdom diameter, and rhabdom cross-sectional area of the rhabdom of *E. brandti* under light and dark adaptation (*p* < 0.01, Table 3).

## 4. Discussion

We found that the compound eye areas and facet numbers of *E. scrobiculatus* adults are significantly larger than those of *E. brandti*. The total area of the compound eye and the number of facets increase with body size, a feature consistent with *Monochamus alternatus* [5]. Larger areas and numbers of facets may indicate wider visual fields, greater sensitivity, and better resolution (acuity) of the compound eyes [26]. Previous studies have shown that the phototropism of *E. scrobiculatus* is significantly higher than *E. brandti* [24], and we speculate that the large compound eye of *E. scrobiculatus* can receive more light in the same condition; hence, they exhibit stronger phototaxis in behavior. 

Both weevils start to climb up the trunks of trees of *A. altissima* to feed on the leaves and branches in daytime [19]. The clear zone in the compound eye is widest in nocturnal beetles and frequently reduced in width or absent in day-active ones [27]. For example, *Aesalus asiaticus* and *Platycerus acuticollis* are daytime-active beetles and lack the clear zone [28]. Unsurprisingly, the present study finds no clear zone between the crystalline cone and the rhabdom in *E. scrobiculatus* and *E. brandti*, and both compound eye types belong to the apposition eye type.

The cornea has the function of protecting the compound eyes of insects from physical damage. Coleoptera insects possess a thicker cornea than Lepidoptera to protect the eye from mechanical damage by hard substances such as soil and grit [29]. The corneal thickness in *Scaphidium japonum* and *Neotriplax lewisi* was approximately 70 µm and 60 µm, respectively [30,31], while *Stigmella microtheriella* adults possess corneal lenses measuring 3.6 μm in thickness [32]. The thickness of the cornea in *E. scrobiculatus* and *E. brandti* is 99.16 ± 1.63 μm and 82.26 ± 0.86 μm, respectively. *E. scrobiculatus* adults are mainly distributed on the ground soil [19], and *E. brandti* adults hide in bark cracks at night [33]. The super thick corneas can protect their eyes from hard objects in the external environment. Under low-light conditions, the thicker cornea can increase the incidence angle of light and improve the sensitivity of the compound eye [34], and *E. scrobiculatus* exhibits stronger phototaxis in behavior. 

Previous studies on insect optical perception showed that the amount of focused energy of the ommatidium is proportional to the size of the cross-sectional area of the ommatidium and the length of the receptors [35]. The length of the rhabdom of *E. scrobiculatus* is significantly greater than that of *E. brandti*. This suggests that the light-focusing ability of *E. scrobiculatus* might be greater than that of *E. brandti*. *E. scrobiculatus* and *E. brandti* have a semi-fused type of rhabdom formed by eight reticular cells (two central cells: R7–R8, surrounded by six peripheral cells: R1–R6). The same kind of rhabdom is also found in *Xanthochroa luteipennis* [36], and it is speculated that their sensitivity to light may be between the open and closed rhabdom types. The research on the mechanism of polarized light perception in insects found that the specialized dorsal rim area of the compound eye is highly suitable for the polarization detection of light [37]. Some insects can rely on the polarized light in the sky to obtain orientation information when lighter sunshine is available. For example, desert ants *Cataglyphis bicolor* have winding routes to forage, but using polarization patterns enables them to return to their nests more quickly along a straight line [38]. Previous studies showed that the direction of numerous microvilli on the rhabdom of dorsal rim omnidia is arranged in two orthogonal directions [39]. *E. scrobiculatus* and *E. brandti* prefer light sources, and only *E. scrobiculatus* can perceive polarized light [24]. However, our study found that the distorted orientation of the rhabdom of *E. scrobiculatus* and *E. brandti* was not aligned in two orthogonal directions. Further transmission electron microscopic observations combined with retinal potential responses are required in the future to reveal whether the two can perceive polarized light.

The compound eyes of *E. scrobiculatus* and *E. brandti* adults are affected by light and dark adaptation, mainly in terms of the movement of pigment granules and changes in the size and shape of the cone cells. The pigment granules are more evenly distributed on the rhabdom in the LA state in some insects, such as *Gryllus bimaculatus* [40], while in the DA state, the pigment granules are primarily distributed in the upper part of the rhabdom. However, the pigment granules are mainly distributed in the area at the junction of the cone and the rhabdom in *E. scrobiculatus* and *E. brandti* under LA conditions, and the pigment granules move longitudinally. They are evenly distributed on both sides of the cone and the optic pole under DA conditions. To control the amount of light reaching the rhabdom, pigment grains migrate within the primary pigment cells to widen or narrow the ommatidial aperture [36]. *E. scrobiculatus* and *E. brandti* have a larger diameter and cross-sectional area at the middle end of the rhabdom during LA than DA. The increase in rhabdom size will widen the receptive field of the photoreceptors, resulting in an increased absolute sensitivity [41]. The cone cells of *E. scrobiculatus* and *E. brandti* are significantly longer in the LA than DA state. The cone cells of *E. scrobiculatus* and *E. brandti* are rounder in the LA than in the DA state. Both *E. scrobiculatus* and *E. brandti* are active during the day and night [19]. The light changes are significant from day to night (evening) or night to day (early morning), and the two beetles possess the ability to adjust the light entering through changes in compound eye structure, including the movement of pigment granules, changes in the size and shape of the cone cells, and the notable expansion of the diameter and cross-sectional area of the rhabdom.

Due to their similarity in body composition, behavior, and resource needs, sympatric and closely related species are frequent competitors. During the competitive process, these species can coexist by niche separation, using distinct resources, or widening the available range to lessen the competitive strain between species. The variations in resource needs across sympatric species are frequently tightly correlated with their physical characteristics. Body type variations will prevent niches from overlapping [42]. For instance, the different beak shapes of European chickadees help prevent feeding niche overlap. These physical variations result from adaptations to various environmental conditions, which support the persistence of niche differentiation [43]. Visual organ structure variations are crucial for niche differentiation, reducing the two species’ fierce interspecific rivalry, facilitating coexistence, and fostering and maintaining species variety. The study on the disparity between the compound eyes of *E. scrobiculatus* and *E. brandti* offers a willing paradigm for investigating niche separation between related species. A fresh line of inquiry into the variations in visual organs offers a solution to the ecological problem of the coexistence of different species. In addition, we recommend using X-ray microtomography (micro-CT) to observe the internal structure of the compound eye in future research. The method provides a way to study the structure of three-dimensional vision without the sectioning damage that comes with traditional microscopy techniques [44] and can deliver the most detailed account of ocellar morphology and fields of view [45,46].

## 5. Conclusions

This study has shown that *E. scrobiculatus* and *E. brandti* adults contain an apposition eye with a semi-fused type of rhabdom. To adapt to the low-light environment, *E. scrobiculatus* possesses a large compound eye area and more ommatidia than *E. brandti*. *E. scrobiculatus* has thicker corneas than *E. brandti*, and the length and cross-sectional area of the rhabdom of *E. scrobiculatus* are more significant than those of *E. brandti*. The two weevils possess different visual organ structures to perceive visual information in the external environment. The study of the compound eye structure of two related species is of great significance to the study of ecological niche differentiation. It provides evidence for exploring the evolution of these species. This experiment lays a foundation for further research on the molecular mechanism of phototaxis.

## Figures and Tables

**Table 1:** Comparison of morphological properties of the compound eyes between *E. scrobiculatus* and *E. brandti*.

Parameters	Unit	*E. scrobiculatus* Female	*E. scrobiculatus* Male	*E. brandti* Female	*E. brandti* Male
Hexagonal facet area	μm^2^	1756.22 ± 46.24 a	1682.06 ± 21.41 a	1405.75 ± 71.55 b	1201.88 ± 42.06 c
irregular facet area	μm^2^	2408.5 ± 67.63 a	2176.29 ± 44.05 b	1599.99 ± 49.25 c	1501.22 ± 20.88 c
Hexagonal facet number	-	387.2 ± 3.93 a	368.2 ± 4.98 b	430.8 ± 7.02 bc	400.6 ± 4.02 c
irregular facet number	-	173.8 ± 3.22 a	186.2 ± 3.56 a	107.2 ± 7.32 b	110.2 ± 5.4 b
Facet number	-	561 ± 7 a	554 ± 5 a	538 ± 11 ab	511 ± 7 b
Compound area	mm^2^	1.1 ± 0.042 a	1.025 ± 0.024 a	0.777 ± 0.043 b	0.647 ± 0.025 b
Hexagonal facet diameter	μm	50.67 ± 1.41 a	52.09 ± 1.43 a	44.14 ± 0.69 b	41.69 ± 0.67 b

Data are expressed as mean ± se. Different letters indicate a significant difference in the same row (*p* < 0.05).

**Table 2:** Comparison of the internal microstructure of the compound eyes between *E. scrobiculatus* and *E. brandti* female adults.

Parameter	Unit	*E. scrobiculatus*	*n*	*E. brandti*	*n*	
Thickness of cornea	μm	99.16 ± 1.63	10	82.26 ± 0.86	10	**
Length of rhabdom	μm	69.12 ± 2.22	10	59.08 ± 2.79	10	*
length of ommatidia	μm	253.94 ± 1.17	10	211.57 ± 3.00	10	**
Interommatidial angle	°	7.08 ± 0.31	5	4.84 ± 0.49	5	**
Diameter of rhabdomere microvilli	μm	2.40 ± 0.20	32	1.76 ± 0.13	30	**

Data are expressed as mean ± se. Means (±se) within a row followed by asterisks indicate significantly different (*t*-test). “*n*” represents the number of samples. “*” indicates *p* < 0.05, “**” indicates *p* < 0.01.

**Table 3:** Changes in the internal microstructure of the compound eye of *E. scrobiculatus* and *E. brandti* female adults in the light/dark adaptation.

Parameter	Unit	LA	*n*	DA	*n*	*p*
Length of the cone cells of *E. scrobiculatus*	μm	9.99 ± 0.38	4	6.74 ± 0.48	4	**
Length of the cone cells of *E. brandti*	μm	7.53 ± 0.40	4	4.50 ± 0.58	4	**
Diameter of rhabdom of *E. scrobiculatus*	μm	24.18 ± 0.2	24	21.21 ± 0.31	24	**
Diameter of rhabdom of *E. brandti*	μm	20.62 ± 0.23	24	19.63 ± 0.17	24	**
Rhabdom cross-sectional area of *E. scrobiculatus*	μm^2^	436.08 ± 6.36	18	372.31 ± 7.96	18	**
Rhabdom cross-sectional area of *E. brandti*	μm^2^	342.66 ± 3.93	12	313.84 ± 4.30	12	**

Data are expressed as mean ± se. Means (±se) within a row followed by asterisks indicate significantly different (*t*-test). “*n*” represents the number of samples. “**” indicates *p* < 0.01.

## Data Availability

The raw data supporting the conclusions of this manuscript will be made available by the authors, without undue reservation, to any qualified researcher.
